# Supplementary material for: Functional anatomy and topographical organization of the frontotemporal arcuate fasciculus
Source: Commun Biol. 2024 Dec 19;7:1655. doi: 10.1038/s42003-024-07274-3 (PMC11659396; doi:10.1038/s42003-024-07274-3)
Supplement: Supplementary file 2 — Supplementary Information [file 42003_2024_7274_MOESM2_ESM.pdf]

Supplementary Information

Methods

1. Data acquisition

1.1.Primary and test-retest datasets (HCP)

Data have been acquired by the Washington University, University of Minnesota and Oxford University (WU-Minn) HCP consortium. Participants recruitment procedures, informed consent, and sharing of de-identified data were approved by the Washington University in St. Louis Institutional Review Board (IRB) [1]

For MRI data acquisition, a custom-made Siemens 3T "Connectome Skyra" (Siemens, Erlangen, Germany), provided with a Siemens SC72 gradient coil and maximum gradient amplitude (Gmax) of 100 mT/m (initially 70 mT/m and 84 mT/m in the pilot phase [2].

For high-resolution T1-weighted MPRAGE scan acquisition, the following parameters were used: voxel size = 0.7 mm, TR = 2400 ms, TE = 2.14 ms [1].

Multi-shell diffusion-weighted imaging (DWI) data (b-values: 1000, 2000, 3000 mm/s^2) were acquired using a single-shot 2D spin-echo multiband Echo Planar Imaging (EPI) sequence. DWI volumes were acquired with 90 directions per shell in addition to 18 non-diffusion-weighted (b = 0 mm/s^2) volumes, and a spatial isotropic resolution of 1.25 mm [3].

Resting-state functional MRI data (rs-fMRI) were acquired with a gradient-echo EPI sequence, using the following parameters: voxel size = 2mm isotropic, TR = 720 ms, TE = 33.1 ms, 1200 frames, ~15 min/run. While data were acquired separately on different days along two different sessions, each session consisting of a left-to-right (LR) and a right-to-left (RL) phase encoding acquisition [1,2,4], the present work features LR and RL acquisitions of the first session only.

1.2. Validation dataset (LEMON)

Data have been acquired by the Max Planck Institute (Leipzig, Germany). The study was carried out in accordance with the Declaration of Helsinki and the study protocol was approved by the ethics committee at the medical faculty of the University of Leipzig. A 3T scanner (MAGNETOM Verio,

Siemens Healthcare GmbH, Erlangen, Germany) equipped with a 32-channel head coil was employed for MRI data acquisition.

The parameters of the MP2RAGE sequence used for structural T1w data acquisition were: voxel size = 1 mm, TR = 5000 ms, TE = 2.92 ms. DWI data (single shell, b = 1000 s/mm^2) were acquired using a multi-band accelerated sequence with spatial isotropic resolution = 1.7 mm, and 60 diffusion-encoding directions plus 7 non-diffusion-weighted (b = 0 s/mm^2) volumes. For rs-fMRI data, a gradient-echo EPI was acquired with the following parameters: phase encoding = AP, voxel size = 2.3 mm isotropic, TR = 1400 ms, TE = 30 ms, 15.30 min/run [5].

2. Data preprocessing

2.1. Structural preprocessing

Skull-stripped T1 weighted images provided by the HCP were segmented into cortical and subcortical gray matter (GM), white matter (WM), and cerebrospinal fluid (CSF) using FAST and FIRST FSL's tools [6,7]. A 5-tissue-type (5TT) image, which was required later for diffusion signal modeling, was obtained from structural segmented images. For the HCP dataset, the already available MNI-space transformations included in the minimal preprocessing pipeline were employed (FLIRT 12 degrees of freedom affine; FNIRT nonlinear registration) [8]. For the LEMON dataset, T1-weighted volumes were also non-linearly registered to the 1-mm resolution MNI 152 asymmetric template using a FLIRT 12 degrees of freedom affine transform and FNIRT non-linear registration [9–11] and direct and inverse transformations were saved; a visual quality check as in Benhajali et al. 2020 was performed to ensure proper alignment of major sulcal and gyral structures.

2.2. DWI preprocessing

The minimal preprocessing pipeline of the HCP data includes eddy currents, EPI distortion and motion correction, and cross-modal linear registration of structural and DWI images [13].

The LEMON DWI scans were preprocessed following the subsequent steps: 1) denoising using Marchenko-Pastur principal component analysis (MP-PCA) [14], 2) removal of Gibbs ringing artifacts

[15], 3) eddy currents, distortion (by exploiting the available reverse-phase encoding scans) and motion correction using EDDY and TOPUP FSL's tools [7,16,17] and 4) bias field correction using the N4 algorithm [18].

2.3. Resting-state fMRI preprocessing

The HCP data minimal preprocessing pipeline included the following steps: 1) artifact and motion correction; 2) registration to 2-mm resolution MNI 152 standard space, 3) high pass temporal filtering (> 2000 s full width at half maximum) [13], 4) denoising, which features ICA-based artifact identification (ICA-FIX) [19] as well as regression of artifacts and motion-related parameters [4]. In addition to the minimal preprocessing, data were band-pass filtered (0.01-0.09 Hz), and the global WM and CSF signal was regressed out to improve ICA-based denoising further [20].

The LEMON dataset processing pipeline included the following steps: 1) removal of the first 5 volumes to allow for signal equilibration, 2) motion and distortion correction, 3) outlier and artifact detection (rapidart) and denoising using component-based noise correction (aCompCor), 4) mean-centering and variance normalization of the time series and 5) spatial normalization to 2-mm resolution MNI 152 standard space [5,21].

To minimize BOLD partial volume sampling from the white matter, both HCP and LEMON rs-fMRI time series were additionally smoothed through convolution with a relatively large Gaussian kernel (6mm full width at half maximum) in line with the reference tw-dFC work [22]. All the additional preprocessing was carried out using CONN toolbox [23].

References

1. Van Essen, D. C. et al. The Human Connectome Project: A data acquisition perspective. Neuroimage 62, 2222–2231 (2012).
2. Uğurbil, K. et al. Pushing spatial and temporal resolution for functional and diffusion MRI in the Human Connectome Project. Neuroimage 80, 80–104 (2013).

3. Sotiropoulos, S. N. et al. Advances in diffusion MRI acquisition and processing in the Human Connectome Project. Neuroimage 80, 125–43 (2013).

4. Smith, S. M. et al. Resting-state fMRI in the Human Connectome Project. Neuroimage 80, 144–168 (2013).

5. Babayan, A. et al. A mind-brain-body dataset of MRI, EEG, cognition, emotion, and peripheral physiology in young and old adults. Sci Data 6, 180308 (2019).

6. Patenaude, B., Smith, S. M., Kennedy, D. N. & Jenkinson, M. A Bayesian model of shape and appearance for subcortical brain segmentation. Neuroimage 56, 907–922 (2011).

7. Smith, S. M. et al. Advances in functional and structural MR image analysis and implementation as FSL. in NeuroImage (2004). doi:10.1016/j.neuroimage.2004.07.051.

8. Glasser, M. F. et al. The minimal preprocessing pipelines for the Human Connectome Project. Neuroimage 80, 105–124 (2013).

9. Andersson, J. L. R., Jenkinson, M., Smith, S. & Andersson, J. FNIRT — FMRIB' Non-Linear Image Registration Tool. Oxford Centre for Functional Magnetic Resonance imaging of the Brain, Department of Clinical Neurology, Oxford University, Oxford, UK (2007).

10. Jenkinson, M. & Smith, S. A global optimisation method for robust affine registration of brain images. Med Image Anal (2001) doi:10.1016/S1361-8415(01)00036-6.

11. Jenkinson, M., Bannister, P., Brady, M. & Smith, S. Improved optimization for the robust and accurate linear registration and motion correction of brain images. Neuroimage (2002).

12. Benhajali, Y. et al. A Standardized Protocol for Efficient and Reliable Quality Control of Brain Registration in Functional MRI Studies. Front Neuroinform 14, 7 (2020).

13. Glasser, M. F. et al. The minimal preprocessing pipelines for the Human Connectome Project. Neuroimage 80, 105–124 (2013).

14. Veraart, J. et al. Denoising of diffusion MRI using random matrix theory. Neuroimage (2016) doi:10.1016/j.neuroimage.2016.08.016.

15. Kellner, E., Dhital, B., Kiselev, V. G. & Reisert, M. Gibbs-ringing artifact removal based on local subvoxel-shifts. Magn Reson Med 76, 1574–1581 (2016).
16. Andersson, J. L. R. & Sotiropoulos, S. N. An integrated approach to correction for off-resonance effects and subject movement in diffusion MR imaging. Neuroimage 125, 1063–1078 (2016).
17. Andersson, J. L. R., Skare, S. & Ashburner, J. How to correct susceptibility distortions in spin-echo echo-planar images: application to diffusion tensor imaging. Neuroimage 20, 870–888 (2003).
18. Tustison, N. J. et al. N4ITK: Improved N3 Bias Correction. IEEE Trans Med Imaging 29, 1310–1320 (2010).
19. Salimi-Khorshidi, G. et al. Automatic denoising of functional MRI data: Combining independent component analysis and hierarchical fusion of classifiers. Neuroimage 90, 449–468 (2014).
20. Plachti, A. et al. Multimodal Parcellations and Extensive Behavioral Profiling Tackling the Hippocampus Gradient. Cerebral Cortex 29, 4595–4612 (2019).
21. Mendes, N. et al. A functional connectome phenotyping dataset including cognitive state and personality measures. Sci Data 6, 180307 (2019).
22. Calamante, F., Smith, R. E., Liang, X., Zalesky, A. & Connelly, A. Track-weighted dynamic functional connectivity (TW-dFC): a new method to study time-resolved functional connectivity. Brain Struct Funct 222, 3761–3774 (2017).
23. Whitfield-Gabrieli, S. & Nieto-Castanon, A. Conn : A Functional Connectivity Toolbox for Correlated and Anticorrelated Brain Networks. Brain Connect 2, 125–141 (2012).

	Neuroscience term	Ventral AF			Middle AF			Dorsal AF		
		r	R^2	p	r	R^2	p	r	R^2	p
Non-linguistic (cognitive)	ambiguous	-0.14	0.02	0.0469	**0.79**	**0.63**	**< 0.001**	**-0.28**	**0.08**	**< 0.001**
	campus	**-0.33**	**0.11**	**< 0.001**	**0.4**	**0.16**	**< 0.001**	**0.33**	**0.11**	**< 0.001**
	consideration	**-0.45**	**0.21**	**< 0.001**	**0.46**	**0.21**	**< 0.001**	**0.39**	**0.15**	**< 0.001**
	gaze	-0.03	0.00	1	**0.51**	**0.26**	**< 0.001**	**-0.32**	**0.10**	**< 0.001**
	locked	**-0.48**	**0.23**	**< 0.001**	**0.22**	**0.05**	**< 0.001**	**0.5**	**0.25**	**< 0.001**
	order	0.18	0.03	0.015	0.13	0.02	0.035	**-0.31**	**0.09**	**< 0.001**
	time	-0.1	0.01	0.2051	**0.25**	**0.06**	**< 0.001**	0.01	0.00	1
	violations	-0.1	0.01	0.2491	**0.74**	**0.55**	**< 0.001**	**-0.3**	**0.09**	**< 0.001**
Non-linguistic (social)	social	**0.38**	**0.14**	**< 0.001**	0.12	0.01	0.018	**-0.59**	**0.35**	**< 0.001**
	social_cognition	**-0.18**	**0.03**	**< 0.001**	**0.68**	**0.46**	**< 0.001**	**-0.19**	**0.03**	**< 0.001**
	social_interaction	**0.29**	**0.08**	**< 0.001**	**0.34**	**0.11**	**< 0.001**	**-0.58**	**0.34**	**< 0.001**
Linguistic (general)	english	0.16	0.02	0.0562	**0.57**	**0.32**	**< 0.001**	**-0.55**	**0.31**	**< 0.001**
	german	-0.06	0.00	1	**0.73**	**0.53**	**< 0.001**	**-0.35**	**0.12**	**< 0.001**
	language	0.09	0.01	0.3998	**0.63**	**0.40**	**< 0.001**	**-0.52**	**0.27**	**< 0.001**
	language_processing	**0.23**	**0.05**	**< 0.001**	**0.49**	**0.24**	**< 0.001**	**-0.61**	**0.37**	**< 0.001**
	linguistic	0.16	0.03	0.0469	**0.57**	**0.33**	**< 0.001**	**-0.55**	**0.31**	**< 0.001**
Linguistic (semantic-comprehension)	comprehension	0.03	0.00	1	**0.71**	**0.50**	**< 0.001**	**-0.46**	**0.21**	**< 0.001**
	meaning	-0.15	0.02	0.0257	**0.79**	**0.63**	**< 0.001**	**-0.25**	**0.06**	**< 0.001**
	noun	**-0.22**	**0.05**	**< 0.001**	**0.75**	**0.56**	**< 0.001**	**-0.14**	**0.02**	**< 0.001**
	semantic	**-0.19**	**0.03**	**< 0.001**	**0.81**	**0.66**	**< 0.001**	**-0.18**	**0.03**	**< 0.001**
	semantic_memory	**-0.29**	**0.08**	**< 0.001**	**0.77**	**0.59**	**< 0.001**	-0.02	0.00	1
	semantic_processing	-0.14	0.02	0.0257	**0.81**	**0.66**	**< 0.001**	**-0.24**	**0.06**	**< 0.001**
Linguistic (phonological-syntactic)	phonological	**0.33**	**0.11**	**< 0.001**	**0.28**	**0.08**	**< 0.001**	**-0.61**	**0.37**	**< 0.001**
	pseudo	**0.4**	**0.16**	**< 0.001**	0.02	0.00	1	**-0.57**	**0.33**	**< 0.001**
	syntactic	0.08	0.01	0.6361	**0.56**	**0.32**	**< 0.001**	**-0.43**	**0.19**	**< 0.001**
	syntax	0.02	0.00	1	**0.66**	**0.44**	**< 0.001**	**-0.42**	**0.18**	**< 0.001**
	sentence	-0.15	0.02	0.0257	**0.8**	**0.64**	**< 0.001**	**-0.29**	**0.09**	**< 0.001**
Linguistic (Reading)	read	0.04	0.00	1	**0.65**	**0.42**	**< 0.001**	**-0.46**	**0.21**	**< 0.001**
	text	**-0.19**	**0.04**	**< 0.001**	**0.78**	**0.61**	**< 0.001**	**-0.21**	**0.05**	**< 0.001**
Acoustic/vocal	pitch	**0.39**	**0.15**	**< 0.001**	**-0.15**	**0.02**	**< 0.001**	**-0.4**	**0.16**	**< 0.001**
	sound	**0.47**	**0.22**	**< 0.001**	**-0.24**	**0.06**	**< 0.001**	**-0.53**	**0.28**	**< 0.001**
	vocal	**0.47**	**0.22**	**< 0.001**	-0.02	0.00	1	**-0.57**	**0.33**	**< 0.001**
	voice	**0.29**	**0.08**	**< 0.001**	**0.37**	**0.14**	**< 0.001**	**-0.5**	**0.25**	**< 0.001**

Supplementary Table 1. Meta-analytic decoding of left AF clusters. For each of the 33 neuroscience terms derived from the meta-analytic screening procedure, Pearson's correlation coefficients (r values) to track-weighted meta-analytic maps as well as their effect size (R^2 coefficient of determination) and spatial autocorrelation-corrected p-values are displayed. All p-values are corrected for multiple comparisons using the Benjamini-Hochberg method.

		Ventral AF			*Middle AF*			*Dorsal AF*		
	**Neuroscience term**	r	R^2	p	r	R^2	p	r	R^2	p
Non-linguistic (cognitive)	ambiguous	-0.03	0.00	1	**0.58**	**0.34**	**< 0.001**	**-0.14**	**0.02**	**< 0.001**
	campus	**-0.53**	**0.28**	**< 0.001**	**0.39**	**0.15**	**< 0.001**	**0.42**	**0.18**	**< 0.001**
	consideration	**-0.52**	**0.27**	**< 0.001**	**0.41**	**0.17**	**< 0.001**	**0.41**	**0.16**	**< 0.001**
	gaze	**-0.14**	**0.02**	**< 0.001**	**0.66**	**0.43**	**< 0.001**	-0.1	0.01	0.0093
	locked	**-0.57**	**0.32**	**< 0.001**	**0.43**	**0.19**	**< 0.001**	**0.43**	**0.18**	**0**
	order	**-0.1**	**0.01**	**< 0.001**	**0.35**	**0.12**	**< 0.001**	**0.16**	**0.02**	**0**
	time	**0.15**	**0.02**	**< 0.001**	**0.24**	**0.06**	**< 0.001**	-0.1	0.01	0.0348
	violations	0.07	0.01	0.1393	**0.67**	**0.44**	**< 0.001**	**-0.3**	**0.09**	**< 0.001**
Non-linguistic (social)	social	-0.07	0.00	0.1349	**0.66**	**0.44**	**< 0.001**	**-0.19**	**0.04**	**< 0.001**
	social_cognition	**-0.41**	**0.17**	**< 0.001**	**0.62**	**0.38**	**< 0.001**	**0.17**	**0.03**	**< 0.001**
	social_interaction	**0.16**	**0.03**	**< 0.001**	**0.59**	**0.34**	**< 0.001**	**-0.35**	**0.12**	**< 0.001**
Linguistic (general)	english	**0.43**	**0.19**	**< 0.001**	**0.21**	**0.04**	**< 0.001**	**-0.45**	**0.20**	**< 0.001**
	german	**0.42**	**0.18**	**< 0.001**	**0.43**	**0.19**	**< 0.001**	**-0.59**	**0.35**	**< 0.001**
	language	**0.28**	**0.08**	**< 0.001**	**0.1**	**0.01**	**< 0.001**	**-0.18**	**0.03**	**< 0.001**
	language_processing	**0.53**	**0.29**	**< 0.001**	0.02	0.00	1	**-0.44**	**0.19**	**< 0.001**
	linguistic	**0.45**	**0.20**	**< 0.001**	**0.23**	**0.05**	**< 0.001**	**-0.46**	**0.21**	**< 0.001**
Linguistic (semantic-comprehension)	comprehension	**0.37**	**0.14**	**< 0.001**	**0.32**	**0.10**	**< 0.001**	**-0.43**	**0.19**	**< 0.001**
	meaning	**0.49**	**0.24**	**< 0.001**	**0.21**	**0.04**	**< 0.001**	**-0.52**	**0.27**	**< 0.001**
	noun	**0.41**	**0.17**	**< 0.001**	**0.35**	**0.12**	**< 0.001**	**-0.52**	**0.27**	**< 0.001**
	semantic	**0.28**	**0.08**	**< 0.001**	**-0.24**	**0.06**	**< 0.001**	-0.06	0.00	0.0348
	semantic_memory	**-0.18**	**0.03**	**< 0.001**	**-0.15**	**0.02**	**< 0.001**	**0.39**	**0.16**	**< 0.001**
	semantic_processing	**0.5**	**0.25**	**< 0.001**	-0.03	0.00	1	**-0.4**	**0.16**	**< 0.001**
Linguistic (phonological-syntactic)	phonological	**0.55**	**0.30**	**< 0.001**	**0.14**	**0.02**	**< 0.001**	**-0.57**	**0.32**	**< 0.001**
	pseudo	**0.57**	**0.33**	**< 0.001**	**-0.19**	**0.04**	**< 0.001**	**-0.47**	**0.22**	**< 0.001**
	syntactic	**0.17**	**0.03**	**< 0.001**	**0.24**	**0.06**	**< 0.001**	-0.09	0.01	0.0422
	syntax	**0.33**	**0.11**	**< 0.001**	**0.44**	**0.19**	**< 0.001**	**-0.41**	**0.17**	**< 0.001**
	sentence	0.13	0.02	0.0186	**0.4**	**0.16**	**< 0.001**	**-0.21**	**0.04**	**< 0.001**
Linguistic (Reading)	read	**0.56**	**0.31**	**< 0.001**	0.07	0.01	0.1393	**-0.53**	**0.28**	**< 0.001**
	text	0.01	0.00	1	**0.47**	**0.22**	**< 0.001**	-0.1	0.01	0.0572
Acoustic/vocal	pitch	**0.41**	**0.17**	**< 0.001**	**0.21**	**0.04**	**< 0.001**	**-0.48**	**0.23**	**< 0.001**
	sound	**0.51**	**0.26**	**< 0.001**	**-0.33**	**0.11**	**< 0.001**	**-0.33**	**0.11**	**< 0.001**
	vocal	**0.57**	**0.33**	**< 0.001**	**0.14**	**0.02**	**< 0.001**	**-0.59**	**0.35**	**< 0.001**
	voice	**0.5**	**0.26**	**< 0.001**	**0.29**	**0.08**	**< 0.001**	**-0.59**	**0.34**	**< 0.001**

Supplementary Table 2. Meta-analytic decoding of right AF clusters. For each of the 33 neuroscience terms derived from the meta-analytic screening procedure, Pearson's correlation coefficients (r values) to track-weighted meta-analytic maps as well as their effect size (R^2 coefficient of determination) and spatial autocorrelation-corrected p-values are displayed. All p-values are corrected for multiple comparisons using the Benjamini-Hochberg method.

**ambiguous**	https://neuroquery.org/query?text=ambiguous
**campus**	https://neuroquery.org/query?text=campus
**comprehension**	https://neuroquery.org/query?text=comprehension
**consideration**	https://neuroquery.org/query?text=consideration
**english**	https://neuroquery.org/query?text=english
**gaze**	https://neuroquery.org/query?text=gaze
**german**	https://neuroquery.org/query?text=german
**language**	https://neuroquery.org/query?text=language
**language_processing**	https://neuroquery.org/query?text=language_processing
**linguistic**	https://neuroquery.org/query?text=linguistic
**locked**	https://neuroquery.org/query?text=locked
**meaning**	https://neuroquery.org/query?text=meaning
**noun**	https://neuroquery.org/query?text=noun
**order**	https://neuroquery.org/query?text=order
**phonological**	https://neuroquery.org/query?text=phonological
**pitch**	https://neuroquery.org/query?text=pitch
**pseudo**	https://neuroquery.org/query?text=pseudo
**read**	https://neuroquery.org/query?text=read
**semantic**	https://neuroquery.org/query?text=semantic
**semantic_memory**	https://neuroquery.org/query?text=semantic_memory
**semantic_processing**	https://neuroquery.org/query?text=semantic_processing
**sentence**	https://neuroquery.org/query?text=sentence
**social**	https://neuroquery.org/query?text=social
**social_cognition**	https://neuroquery.org/query?text=social_cognition
**social_interaction**	https://neuroquery.org/query?text=social_interaction
**sound**	https://neuroquery.org/query?text=sound
**syntactic**	https://neuroquery.org/query?text=syntactic
**syntax**	https://neuroquery.org/query?text=syntax
**text**	https://neuroquery.org/query?text=text
**time**	https://neuroquery.org/query?text=time
**violations**	https://neuroquery.org/query?text=violations
**vocal**	https://neuroquery.org/query?text=vocal
**voice**	https://neuroquery.org/query?text=voice

Supplementary Table 3. Links to the publications related to the meta-analytic terms.
