# Supplementary material for: Functional anatomy and topographical organization of the frontotemporal arcuate fasciculus
Source: Commun Biol. 2024 Dec 19;7:1655. doi: 10.1038/s42003-024-07274-3 (PMC11659396; doi:10.1038/s42003-024-07274-3)
Supplement: Supplementary file 3 — Reporting Summary [file 42003_2024_7274_MOESM3_ESM.pdf]

nature portfolio

Corresponding author(s): Alberto Cacciola

Last updated by author(s): 13/9/24

Reporting Summary

Nature Portfolio wishes to improve the reproducibility of the work that we publish. This form provides structure for consistency and transparency in reporting. For further information on Nature Portfolio policies, see our Editorial Policies and the Editorial Policy Checklist.

Statistics

For all statistical analyses, confirm that the following items are present in the figure legend, table legend, main text, or Methods section.

n/a	Confirmed	
☐	☒	The exact sample size (*n*) for each experimental group/condition, given as a discrete number and unit of measurement
☐	☒	A statement on whether measurements were taken from distinct samples or whether the same sample was measured repeatedly
☐	☒	The statistical test(s) used AND whether they are one- or two-sided<br>*Only common tests should be described solely by name; describe more complex techniques in the Methods section.*
☐	☒	A description of all covariates tested
☐	☒	A description of any assumptions or corrections, such as tests of normality and adjustment for multiple comparisons
☐	☒	A full description of the statistical parameters including central tendency (e.g. means) or other basic estimates (e.g. regression coefficient) AND variation (e.g. standard deviation) or associated estimates of uncertainty (e.g. confidence intervals)
☐	☒	For null hypothesis testing, the test statistic (e.g. *F*, *t*, *r*) with confidence intervals, effect sizes, degrees of freedom and *P* value noted<br>*Give P values as exact values whenever suitable.*
☒	☐	For Bayesian analysis, information on the choice of priors and Markov chain Monte Carlo settings
☒	☐	For hierarchical and complex designs, identification of the appropriate level for tests and full reporting of outcomes
☐	☒	Estimates of effect sizes (e.g. Cohen's *d*, Pearson's *r*), indicating how they were calculated

Our web collection on statistics for biologists contains articles on many of the points above.

Software and code

Policy information about availability of computer code

Data collection	Data from the publicly available Human Connectome Project (HCP) and the Leipzig Study for Mind-Body-Emotion Interactions (LEMON) datasets.
Data analysis	open software FSL6.0, MRtrix3, CONN toolbox, TractSeg, GIFT toolbox, Neuroquery image search tool.<br>The code and maps obtained in the present work will be available after publication at https://github.com/BrainMappingLab.

For manuscripts utilizing custom algorithms or software that are central to the research but not yet described in published literature, software must be made available to editors and reviewers. We strongly encourage code deposition in a community repository (e.g. GitHub). See the Nature Portfolio guidelines for submitting code & software for further information.

Data

Policy information about availability of data

All manuscripts must include a data availability statement. This statement should provide the following information, where applicable:

- Accession codes, unique identifiers, or web links for publicly available datasets
- A description of any restrictions on data availability
- For clinical datasets or third party data, please ensure that the statement adheres to our policy

The code and maps obtained in the present work will be available after publication at https://github.com/BrainMappingLab. The structural and functional MRI data are available on the HCP (https://www.humanconnectome.org/) and LEMON (http://fcon_1000.projects.nitrc.org/indi/retro/MPI_LEMON.html) websites.

Research involving human participants, their data, or biological material

Policy information about studies with human participants or human data. See also policy information about sex, gender (identity/presentation), and sexual orientation and race, ethnicity and racism.

Reporting on sex and gender	Two HCP datasets have been employed for the present work: the first dataset (primary dataset) consisted of 210 healthy participants (males=92, females=118), and the second dataset (test-retest dataset) included 44 participants with available test-retest MRI scans (males = 13; females = 31).<br>A validation dataset of 213 healthy subjects (males=138, females=75) were retrieved from the LEMON database.<br>No sex-related analysis was performed as deemed not of interest for the aim of the work.
Reporting on race, ethnicity, or other socially relevant groupings	As per HCP and LEMON databases.
Population characteristics	Two HCP datasets have been employed for the present work: the first dataset (primary dataset) consisted of 210 healthy participants (males=92, females=118, age range 22-36 years), and the second dataset (test-retest dataset) included 44 participants with available test-retest MRI scans (males = 13; females = 31; age range: 22–36 years).<br>A validation dataset including high-quality structural, diffusion, and rs-fMRI data of 213 healthy subjects (males=138, females=75, age range 20-70 years) were retrieved from the LEMON database.
Recruitment	The primary dataset (HCP) was provided by the Human Connectome Project, WU-Minn Consortium (Principal Investigators: David Van Essen and Kamil Ugurbil; 1U54MH091657). The data are openly available from https://www.humanconnectome.org/<br>The "Leipzig Study for Mind-Body-Emotion Interactions" (LEMON) data used as a validation dataset was provided by the Mind-Body-Emotion group at the Max Planck Institute for Human Cognitive and Brain Sciences. The data are openly available from http://fcon_1000.projects.nitrc.org/indi/retro/MPI_LEMON.html.
Ethics oversight	HCP: Participants recruitment procedures, informed consent, and sharing of de-identified data were approved by the Washington University in St. Louis Institutional Review Board (IRB).<br>LEMON: The study was carried out in accordance with the Declaration of Helsinki and the study protocol was approved by the ethics committee at the medical faculty of the University of Leipzig.

Note that full information on the approval of the study protocol must also be provided in the manuscript.

Field-specific reporting

Please select the one below that is the best fit for your research. If you are not sure, read the appropriate sections before making your selection.

☒ Life sciences ☐ Behavioural & social sciences ☐ Ecological, evolutionary & environmental sciences

For a reference copy of the document with all sections, see nature.com/documents/nr-reporting-summary-flat.pdf

Life sciences study design

All studies must disclose on these points even when the disclosure is negative.

Sample size	Two HCP datasets have been employed for the present work: the first dataset (primary dataset) consisted of 210 healthy participants (males=92, females=118, age range 22-36 years), and the second dataset (test-retest dataset) included 44 participants with available test-retest MRI scans (males = 13; females = 31; age range: 22–36 years).<br>A validation dataset including high-quality structural, diffusion, and rs-fMRI data of 213 healthy subjects (males=138, females=75, age range 20-70 years) were retrieved from the LEMON database.<br>Structural and functional MRI with >40 participants is considered standard practice in the field. Using three different datasets help demonstrate the methods/analysis's validity and interest. for the HCP test-retest and LEMON datasets, the number of participants corresponded to all the data available in the datasets.
Data exclusions	There is no data exclusion.
Replication	A validation dataset including high-quality structural, diffusion, and rs-fMRI data of 213 healthy subjects (males=138, females=75, age range 20-70 years) were retrieved from the LEMON database. We decided to keep the preprocessing pipelines different, as in previous work, to further highlight the reproducibility of our findings.<br>The components identified by group ICA showed very high out-of-sample reproducibility (> 0.90), suggesting that they may capture functional features of the arcuate fasciculus that are robust to experimental differences in data acquisition and processing.
Randomization	No randomization was needed for the design of our analysis.
Blinding	This is a data-driven study; raw data are available as part of the HCP and LEMON datasets; the results of the study are available for replication; therefore blinding was not relevant for this study.

Reporting for specific materials, systems and methods

We require information from authors about some types of materials, experimental systems and methods used in many studies. Here, indicate whether each material, system or method listed is relevant to your study. If you are not sure if a list item applies to your research, read the appropriate section before selecting a response.

Materials & experimental systems

n/a	Involved in the study
☒	☐ Antibodies
☒	☐ Eukaryotic cell lines
☒	☐ Palaeontology and archaeology
☒	☐ Animals and other organisms
☒	☐ Clinical data
☒	☐ Dual use research of concern
☒	☐ Plants

Methods

n/a	Involved in the study
☒	☐ ChIP-seq
☒	☐ Flow cytometry
☐	☒ MRI-based neuroimaging

Plants

Seed stocks

Report on the source of all seed stocks or other plant material used. If applicable, state the seed stock centre and catalogue number. If plant specimens were collected from the field, describe the collection location, date and sampling procedures.

Novel plant genotypes

Describe the methods by which all novel plant genotypes were produced. This includes those generated by transgenic approaches, gene editing, chemical/radiation-based mutagenesis and hybridization. For transgenic lines, describe the transformation method, the number of independent lines analyzed and the generation upon which experiments were performed. For gene-edited lines, describe the editor used, the endogenous sequence targeted for editing, the targeting guide RNA sequence (if applicable) and how the editor was applied.

Authentication

Describe any authentication procedures for each seed stock used or novel genotype generated. Describe any experiments used to assess the effect of a mutation and, where applicable, how potential secondary effects (e.g. second site T-DNA insertions, mosiacism, off-target gene editing) were examined.

Magnetic resonance imaging

Experimental design

Design type

Resting-state

Design specifications

Fully described on HCP (https://www.humanconnectome.org/) and LEMON websites (http://fcon_1000.projects.nitrc.org/indi/retro/MPI_LEMON.html)

Behavioral performance measures

As per HCP and LEMON procedures.

Acquisition

Imaging type(s)

Structural, diffusion and functional

Field strength

3T

Sequence & imaging parameters

Structural HCP
T1-weighted MPRAGE scan acquisition, the following parameters were used: voxel size = 0.7 mm, TR = 2400 ms, TE = 2.14 ms.

Structural LEMON
The parameters of the MP2RAGE sequence used for structural T1w d
ata acquisition were: voxel size = 1 mm, TR = 5000ms, TE = 2.92ms.

Diffusion HCP
Multi-shell diffusion-weighted imaging (DWI) data (b-values: 1000, 2000, 3000 mm/s2) were acquired using a single-shot 2D spin-echo multiband Echo Planar Imaging (EPI) sequence. DWI volumes were acquired with 90 directions per shell in addition to 18 non-diffusion-weighted (b = 0 mm/s2) volumes, and a spatial isotropic resolution of 1.25 mm.

Diffusion LEMON
DWI data (single shell, b = 1000 s/mm2) were acquired using a multi-band accelerated sequence with spatial isotropic resolution = 1.7 mm, and 60 diffusion-encoding directions plus 7 non-diffusion-weighted (b = 0 s/mm2) volumes.

rs-fMRI HCP

Acquired with a gradient-echo EPI sequence, using the following parameters: voxel size = 2mm isotropic, TR = 720 ms, TE = 33.1 ms, 1200 frames, ~15 min/run. While data were acquired separately on different days along two different sessions, each session consisting of a left-to-right (LR) and a right-to-left (RL) phase encoding acquisition, the present work features LR and RL acquisitions of the first session only.

rs-fMRI LEMON
A gradient-echo EPI was acquired with the following parameters: phase encoding = AP, voxel size = 2.3mm isotropic, TR = 1400ms, TE = 30ms, 15.30 min/run.

Area of acquisition: Whole brain

Diffusion MRI ☒ Used ☐ Not used

Parameters: Diffusion HCP
Multi-shell diffusion-weighted imaging (DWI) data (b-values: 1000, 2000, 3000 mm/s2) were acquired using a single-shot 2D spin-echo multiband Echo Planar Imaging (EPI) sequence. DWI volumes were acquired with 90 directions per shell in addition to 18 non-diffusion-weighted (b = 0 mm/s2) volumes, and a spatial isotropic resolution of 1.25 mm.

Diffusion LEMON
DWI data (single shell, b = 1000 s/mm2) were acquired using a multi-band accelerated sequence with spatial isotropic resolution = 1.7 mm, and 60 diffusion-encoding directions plus 7 non-diffusion-weighted (b = 0 s/mm2) volumes.

Preprocessing

Preprocessing software: The MRI data used in this study were preprocessed data from the HCP and LEMON databases. The LEMON DWI scans were available only in raw form and were preprocessed entirely with a dedicated pipeline included in the MRtrix3 software.

Normalization: For the HCP dataset, the already available MNI-space transformations included in the minimal preprocessing pipeline were employed (FLIRT 12 degrees of freedom affine; FNIRT nonlinear registration). For the LEMON dataset, T1-weighted volumes were also non-linearly registered to MNI 152 template using a FLIRT 12 degrees of freedom affine transform and FNIRT non-linear registration and direct and inverse transformations were saved

Normalization template: MNI 152 2mm isotropic

Noise and artifact removal: As per the HCP and LEMON pipelines

Volume censoring: As per the HCP and LEMON pipelines

Statistical modeling & inference

Model type and settings: Bundle-specific tw-dFC volumes underwent a spatial group ICA framework implemented in the Group ICA of FMRI Toolbox (GIFT). To select the most appropriate number of components (k) for arcuate fasciculus (AF) parcellation given our data, the ICA pipeline was iterated for the left and right AF separately at different values of k, ranging from 2 to 5, and for each ICA solution and different measures were calculated. The optimal number of components was decided based on a consensus approach between all these metrics: the value k for which most metrics showed the highest value. To derive a parcellation of the AF from the component maps, we applied a k-means clustering procedure in the component space. To provide a functional characterization for the white matter clusters derived from AF parcellation, we employed a custom meta-analytic approach, specifically designed to account both for the discrete nature of binary clusters and the white matter nature of the underlying spatial maps.

Effect(s) tested: N/A

Specify type of analysis: ☐ Whole brain ☐ ROI-based ☒ Both

Anatomical location(s): For bundle-specific tractography of the arcuate fasciculus TractSeg (https://github.com/MIC-DKFZ/TractSeg/), a convolutional neural network-based tract segmentation approach, was employed.

Statistic type for inference: N/A

(See Eklund et al. 2016)

Correction: Pairwise Pearson's correlation was employed to quantify the similarity between each cluster distance map and the resulting track-weighted term maps. To address the spatial autocorrelation (SA) properties of arcuate maps, statistical significance was assessed using a permutational approach described in Burt et al. (2020). This approach involved the generation of SA-preserving surrogated maps through 1000 permutations. The resulting p-values underwent correction for multiple comparisons using the Benjamini-Hochberg method.

Models & analysis

n/a	Involved in the study
☐	☒ Functional and/or effective connectivity
☒	☐ Graph analysis
☒	☐ Multivariate modeling or predictive analysis

Functional and/or effective connectivity

Track-weighted dynamic functional connectivity (tw-dFC) analysis as in doi: 10.1007/s00429-017-1431-1.
